# Microbial Detoxification of Residual Pesticides in Fermented Foods: Current Status and Prospects

**DOI:** 10.3390/foods12061163

**Published:** 2023-03-09

**Authors:** Nadya Armenova, Lidia Tsigoriyna, Alexander Arsov, Kaloyan Petrov, Penka Petrova

**Affiliations:** 1 Institute of Chemical Engineering, Bulgarian Academy of Sciences, 1113 Sofia, Bulgaria; 2 Institute of Microbiology, Bulgarian Academy of Sciences, 1113 Sofia, Bulgaria

**Keywords:** pesticide, food, lactic acid bacteria, food detoxification

## Abstract

The treatment of agricultural areas with pesticides is an indispensable approach to improve crop yields and cannot be avoided in the coming decades. At the same time, significant amounts of pesticides remain in food and their ingestion causes serious damage such as neurological, gastrointestinal, and allergic reactions; cancer; and even death. However, during the fermentation processing of foods, residual amounts of pesticides are significantly reduced thanks to enzymatic degradation by the starter and accompanying microflora. This review concentrates on foods with the highest levels of pesticide residues, such as milk, yogurt, fermented vegetables (pickles, kimchi, and olives), fruit juices, grains, sourdough, and wines. The focus is on the molecular mechanisms of pesticide degradation due to the presence of specific microbial species. They contain a unique genetic pool that confers an appropriate enzymological profile to act as pesticide detoxifiers. The prospects of developing more effective biodetoxification strategies by engaging probiotic lactic acid bacteria are also discussed.

## 1. Introduction

Pesticides are biological or chemical substances intended for preventing, destroying, or controlling any pest that causes losses in agricultural and food production (raw materials and food), processing, storage, or marketing. Depending on their use, several types are known: fungicides (prevent the development of molds), insecticides (destroy insects), herbicides (used against weeds), and pesticides (repel or destroy rodents, nematodes, and mollusks) [1,2].

Although pesticides solve significant agricultural problems in weed and pest control, pesticide residues are released into the environment, especially newer pesticides, which are water soluble. The older types of pesticides, such as DDT, are less soluble in water but tend to remain in the soil for a long time. That is why, under the coordination of the European Food Safety Authority (EFSA), annual reports of the European Union (EU) monitor the pesticide residues in water and food [3]. The maximum residue level (MRL) is an important determinant of human health risk. Pesticide residue levels in food are subject to legal regulation to minimize their harmful effects [4]. However, in many developing countries, such legislation has not been introduced or is poorly enforced [5]. MRLs are affected by food processing including fermentation, heat treatment, and drying. In addition, the chemical nature of pesticides and some factors, such as pH, light, metal ions, and ozone, also affect the degradation of pesticide residues [6,7] Although MRLs are a reliable and useful tool for regulating the use of pesticides, they are not sufficient to assess human health risks unless the amounts of residues that remain after food processing are also estimated [8]. The MRL for drinking water regulated by Directive 98/83 of the European Council is set at 0.1 μg/L for each pesticide or its metabolite, except for aldrin, dieldrin, heptachlor, and heptachlor epoxide where the limit is 0.03 μg/L. The limit for the sum of identified and quantified pesticides and their metabolites is 0.5 μg/L [9]. The MRL values for different foods and pesticides are annually updated by the European Commission [10].

**Figure 1:**
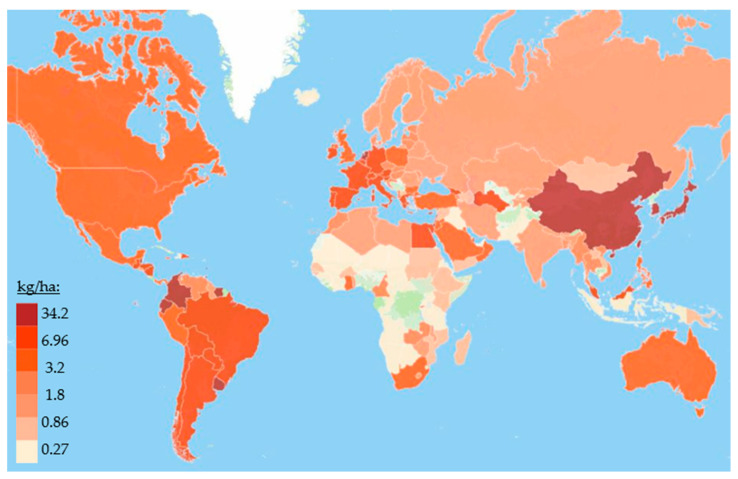
Use of pesticides per area of cropland in 2019, according to FAO statistics [12].

Pesticide use is widespread worldwide (Figure 1). Approximately 3 billion kg are applied annually, as over 500 compounds are registered and used as pesticides or pesticide metabolites [11]. According to the Food and Agriculture Organization of the United Nations (FAO) statistics for 2019, in some countries, pesticide use exceeded 34 kg per hectare of the cultivated area [12].

Compared to previous analyzed periods (2014 to 2017), even with the strict regulations of the EU, in 2020 pesticide residues in foods increased. The twelve most consumed foods by EU citizens contain pesticides in concentrations above the MRL and these are carrots, cauliflower, kiwi, onions, oranges, pears, potatoes, beans, brown rice, rye grain, beef liver, and poultry fat [10]. Unprocessed fruits grown in the EU contain 13–14 different pesticides, and among the goods with the highest frequency of detected pesticides above the norm is wine. Dimethoate, linuron, and cypermethrin are most often found in oranges; triadimenol—in dried nuts; iprodione, linuron, dieldrin, and chlorpyrifos-methyl—in carrots; chlorpyrifos, fipronil, and diphenylamine—in potatoes; and thiacloprid—in rye flour and dough. Hexachlorobenzene, which is used as a fungicide in poultry feed storage, finally accumulates in poultry fat. Apparently, moving pesticides from soil to crops is easy because soil particles adhere to the plant surface [13]. Pesticide uptake also occurs via roots and through the vapor; therefore, high temperature, high wind speed, and low humidity lead to increased uptake of pesticides in plants. The soil contamination risk is higher in root crops and leafy vegetables [14].

Physical and chemical methods are known to reduce the residual amount of pesticides in food, for example, water washing, hydro cooling, brushing, electrolyzed water treatment, boiling, trimming, peeling, cooking, ozonation, drying and dehydration, pasteurization, canning of fruits and vegetables, bleaching, and oil deodorizing [15,16,17,18].

These methods certainly reduce the content of pesticides in food, but some of them are only somewhat effective and others are expensive, which is why current scientific interest is focused on the possibility of pesticide detoxification through fermentation in the food processing process. Microbial detoxification is being established as the most cost-effective approach to combat unavoidable pesticide contamination. Local microflora available in foods or purposefully added probiotic strains can metabolize a wide range of synthetic insecticides and use them as a source of carbon and energy [19]. The undisputed leaders in this process are the lactic acid bacteria (LAB) [20], but of course, many other species contribute to the purification of food as a result of fermentation, such as yeasts, molds, and representatives of many other microbial groups. This review examines the subtle interactions between microorganisms and particular pesticide substances. A detailed overview of the microbial species and strains involved in the detoxification process, their enzymatic spectrum, and capabilities that contribute to making the food a safe-to-eat product is provided below.

## 2. Overview of Pesticides in Food

**Figure 2:**
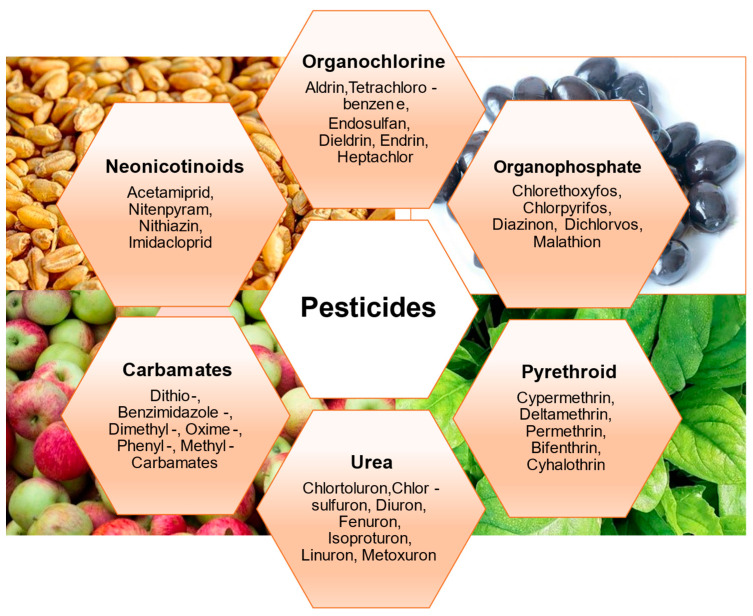
Pesticides of the different classes most frequently found in food.

There is a vast range of pesticides available on the market. Over 1200 active substances have been registered for the production of pesticides. Pesticides can be classified into more than 100 classes/groups, of which the most important and widely applied are organochlorines, organophosphates, carbamates, triazines, pyrethroids, phenoxy alkane, and glyphosate-based pesticides. The most frequently used pesticides usually found in foods are shown in Figure 2.

### 2.1. Organochlorine Pesticides (OCP)

OCPs are stable and very persistent in the environment. When taken into the human body, organochlorines are capable of accumulating in the adipose tissue with possible long-term effects; they also affect the central nervous system, altering the electrophysiological properties of membranes [21]. Because of their anti-estrogenic effects, OCPs are classified as endocrine disruptors [22]. The oldest organochlorine is the insecticide DDT (1,1,1-trichloro-2,2-bis (4-chlorophenyl) ethane), applied since 1939 and already prohibited in many countries. Structurally, organochlorines are divided into five classes: (1) DDT and its main aerobic metabolite DDE (2,2-bis (4-chlorophenyl)-1,1,1-dichloroethylene); (2) HCH (hexachlorocyclohexane), e.g., lindane; (3) cyclodienes: aldrin, dieldrin, endrin, heptachlor, chlordane, endosulfan; (4) toxaphene; (5) mirex and chlordecone. The field half-life of aldrin is 365 days, and of DDT—up to 30 years in soils; that is why DDT has been detected widely in environments and biological samples [23]. In food, OCPs were detected in milk, hen eggs, and breast milk [22]; in fruit and vegetable samples, such as in leafy vegetables (parsley and watercress), heptachlor is the most persistent member [24]. Another study found OCP residues in 11 types of vegetable oils: olive, corn, colza, camellia, peanut, soybean, linseed, blend, sunflower, and rice, with the highest concentration of OCPs found in sesame oil samples [25]. Multiple OCPs pesticide residues above the MRLs (HCHs; Drins; Heptachlor; Chlordane; DDT) were found in vegetable samples of tomato, cabbage, cucumber, carrot, eggplant, watermelon, and lettuce [26,27,28]. In China, OCP contaminations of Chinese cabbage and Welsh onion mainly originate from new inputs of lindane, while eggplant, pepper, cucumber, and radish accumulate historical residues of lindane in soil [28]. The milk samples containing residues of DDT, DDE, dieldrin, Ɣ-HCH, α-endosulfan, β-endosulfan, and endosulfan sulfate are derived from animals contaminated by feeding [29,30]. Data about residual amounts of OCP found in foods and grains are presented in Table 1.

### 2.2. Organophosphate Pesticides (OPP)

OPPs are esters of phosphoric acid effective as insecticides, acaricides, and miticides, commonly applied to treat stored cereal grains. Most often used are diazinon, chlorpyrifos, chlorpyrifos-methyl, phorate, dimethoate, malathion, acephate, azinphos-methyl, phosmet, dicrotophos, and naled [47]. In general, OPPs are acutely toxic to bees, wildlife, and humans. They are acetylcholinesterase inhibitors, and the intoxication symptoms include coma, dizziness, nausea, headache, cramps, convulsions, loss of reactions, and even death [48,49,50]. The systemic herbicide glyphosate (N-(phosphonomethyl) glycine) is another organophosphorus compound (phosphonate), but it acts by inhibiting the plant enzyme 5-enolpyruvylshikimate-3-phosphate synthase [51].

The OPP residues can be detected in fruits, dairy products, cereals, olives, and vegetables (Table 1). In the EU, glyphosate residues are most frequently found in dry lentils, linseeds, soya beans, dry peas, tea, buckwheat, barley, wheat, and rye in concentrations around the MRL of 0.5 mg/kg. A survey in Canada, however, found up to 4 mg/kg glyphosate in beans and chickpeas, many times higher than the Canadian MRL of 0.1 mg/kg [52].

### 2.3. Pyrethroids

These are natural insecticides derived from pyrethrum extracts of chrysanthemum flowers, but also large quantities of synthetic pyrethroids are made; they possess low toxicity to birds and mammals, high toxicity to arthropods and fish, and are ineffective against underground pests. About 30% of fruits and 25% of vegetables on the Chinese market contain fipronil residues, with the highest concentrations in litchi and in leaf lettuce [32,53,54]. Residues of cypermethrin, deltamethrin, and fenpropathrin were detected in seafood in China; fenpropathrin concentrations exceeding the Japanese limit standard were detected also in mollusks, crustaceans, and fish [55].

Pyrethroids with concentrations exceeding MRL were detected in nine leafy vegetables such as Chinese cabbage, baby Chinese cabbage, pakchoi, spinach, celery, *Brassica parachinensis* Bailey, romaine lettuce, and mater convolvulus, collected during 2017–2019 in China [32]. The most frequently spread pesticides were cypermethrin and λ-cyhalothrin. Exceeding the MRL values were found in Chinese cabbage (λ-cyhalothrin and bifenthrin) and pakchoi (cypermethrin) samples. Pyrethroid residues in high concentrations have been found also in fruits, vegetables, tea, and honey [32]. In leafy vegetable samples, pakchoi, choy sum, head mustard, and leaf mustard were detected as containing cypermethrin, deltamethrin, and λ- cyhalothrin above the maximum residue limit for Vietnam [41].

### 2.4. Urea Pesticides

Phenyl urea derivatives (PUHs) are used as herbicides for weed control on crops such as beans, maize, fruit, and wheat [56]. In this group are fall chlortoluron, chlorsulfuron, linuron, diuron, fenuron, isoproturon, and many others. They possess moderate toxicity to humans and animals by altering calcium metabolism and bone morphology [57]. In spite of the fact that some of the urea pesticides are affiliated with the EU “black list” of dangerous compounds, diuron, monuron, and linuron have been found in concentrations higher than MRL in fruit juices (orange, strawberry, cherry, and apple) [58], corn, rice [59], courgette cucumbers, lettuce, peppers [60], fresh and processed tomatoes [61], etc.

### 2.5. Carbamates

Carbamate pesticides are esters of carbamic acid used as insecticides, fungicides, selective herbicides, and acaricides in the production of fruits, vegetables, hops cultures, grains, or for seed treatment. Widely used are thiobencarb, propoxur, molinate, disulfiram, pyridostigmine, methiocarb, and carbaryl. They also act as ACE inhibitors, although they are generally shorter lived than OPPs. Human acute poisoning is fairly common and severe, with symptoms such as bradycardia, blurred vision, nausea, vomiting, cough, wheezing, slurred speech, drowsiness, and muscle cramps [62]. In food, carbaryl was detected in spinach crops in Mexico with concentrations up to 0.399 mg/kg (as the EU MRL is 0.01 mg/kg) [36], kresoxim (0.18 mg/kg), and thiodicarb (0.038 mg/kg) in potato tubers in Egypt [39].

Carbofuran is one of the most toxic carbamate pesticides, classified by the WHO in the category of highly hazardous insecticides class Ib, and that is why it has been banned in Canada and the European Union since 2008. However, carbofuran residues exceeding the MRL have been detected in 61 different types of fruits and vegetables collected from Chinese markets: wolfberry leaves, nectarines, cowpeas, strawberries, tangerines, Chinese cabbage, guava, and snap beans [44]. Carbaryl and carbofuran were found in high concentrations in Cameroon, the most contaminated foods being pepper, soybeans, Egusi seeds, maize, and groundnuts [30]. The notorious record value reported for carbofuran pollution is 1.66 mg/kg in potatoes [40].

### 2.6. Neonicotinoids

Neonicotinoids are nerve-paralytic insecticides that are chemically similar to nicotine, including acetamiprid, clothianidin, dinotefuran, imidacloprid, nitenpyram, nithiazine, thiacloprid, and thiamethoxam. It is a relatively new family of pesticides (since 1990) that is rapidly replacing the use of organophosphates, carbamates, pyrethrins, and pyrethroids due to their lower toxicity to birds and mammals [63]. However, the use of neonicotinoids is risky for human health and beneficial insects (such as bees). In humans, exposure results in neurological damage, especially when it occurs during the embryonic period; leads to cognitive and memory impairments; impairs neuronal development, with a reduction in neurogenesis; and induces neuroinflammation. In food, high concentrations of neonicotinoids were found in honey [64], as well as in farmed algae, fish, and shrimp [65]. Low amounts of acetamiprid, thiamethoxam, clothianidin, imidacloprid, and thiacloprid were found in Swiss cow, goat, and sheep milk, as well as in human breast milk [66]. Seven neonicotinoids were detected in cucumbers, six in eggplant and cabbage, and five in tomatoes, kidney beans, carrots, Chinese greens, and apples [67]. Recently, Montiel-León et al. detected the neonicotinoid insecticides imidacloprid, acetamiprid, and clothianidin in lettuce, apples, grapes, and tomatoes [39].

## 3. Microbial Detoxification of Fermented Foods Containing High Amounts of Pesticides

The scientific research related to microbial degradation of pesticide residues began in the 1940s when people began to pay more attention to environmental protection [68]. Biodegradation is the use of microorganisms or their enzymes to degrade and detoxify xenobiotics in food, water, and soil. The method is an efficient and inexpensive option to deal with pesticide pollution [69,70]. Ideally, the result should be complete mineralization/degradation of the pesticide to H_2_O and CO_2_ without the accumulation of more toxic intermediates [71]. Pesticides can be degraded metabolically by microorganisms. In catabolite degradation, microorganisms use pesticides as the main source of energy (as a carbon, nitrogen, or phosphorus source). Co-metabolic degradation occurs when they are not used as a primary energy source [72].

The reduction of pesticide residues during fermentation has been studied in various food products [72,73,74,75,76,77]. Microorganisms with the highest pesticide degradation activity belong to the genera *Bacillus, Micrococcus, Arthrobacter, Corynebacterium, Flavobacterium, Pseudomonas*, and *Rhodococcus*, as well as fungi from the genera *Penicillium, Aspergillus, Fusarium*, and *Trichoderma* [78,79,80]. Although fungal bioremediation of pesticides has significant potential, it has received less attention than bacterial bioremediation. However, most of the soil bacterial isolates are not applicable for food detoxification because of their pathogenic nature.

### 3.1. Milk and Yogurt

A number of comprehensive studies have shown that strains of different LAB species possess the natural ability to degrade pesticides in vitro and alleviate pesticide poisoning in vivo [81,82,83]. The first studies of the natural degradation of pesticides in dairy products due to the action of autochthonous microflora date back to the 1960s with the works of Kallman and Andrews [84], investigating organochlorine residues. They reported that the conversion of DDT by yeast occurs rather because of a pH decrease. Then, a true degradation of DDT (1 mg/L in milk and cheese) was shown by Abou-Arab [73], who observed the activity of starters containing *Lactobacillus delbrueckii* subsp. *bulgaricus, Streptococcus thermophilus*, and yeasts. The maximum reduction in total DDT of contaminated Ras cheese and milk was achieved after 8 days, 10 days, and 7 days for streptococci, lactobacilli, and yeasts, respectively [73]. The achieved reduction of pesticide levels is shown in Table 2.

Much later, *Latilactobacillus sakei* strain pro7 reached 95.1% biodegradation of DDT with a concentration of 20 mg/kg [91]. Duan et al. [85] proved that starter cultures (*L acidophilus*, *L. delbrueckii* subsp. *bulgaricus*, *Lp. plantarum*, *Lp. rhamnosus*, *Lc. casei*, *S. thermophilus,* and *Bif. bifidum*) decrease the concentration of five different OCPs during yogurt and cheese production. Witczak and Mituniewicz-Małek [86] demonstrated a significant reduction of the level of organochlorine pesticide residues after 14 days in cold storage by the addition of a probiotic mixture of *L. acidophilus* LA-5 and *Bif. animalis* subsp. *lactis* BB-12 to the yogurt starter cultures. The most significant was the reduction of heptachlor—by 36.6%.

Considering OPP reduction, Zhou and Zhao [88] showed the effectiveness of five different species of LAB for the elimination of organophosphorus pesticides in skimmed milk. Due to the activity of LAB, OPP concentrations decreased by 7.0–64.6%. All nine investigated compounds were most susceptible to *L. delbrueckii* subsp. *bulgaricus*, which increased their degradation rate constants by 18.3–133.3%. Zhang et al. [89] tested ten LAB strains (Table 2) and four combinations of strains for the degradation of five organophosuphate pesticides in skimmed milk. *Lev. brevis* 1.0209 was found to possess the highest pesticide degradation activity. Similar results were obtained by Zhao and Wang [90], reporting a strong acceleration of OPP degradation in skimmed milk by *L. delbrueckii* subsp. *bulgaricus*, *Lc. paracasei*, and *Lp. plantarum*. They added seven organophosphate pesticides to milk samples and 24 h later observed reduced pesticide concentrations by 20.9% (of methyl parathion/methyl parathion incubated with *Lc. paracasei*), and by 46.9% (of malathion/malathion incubated with *Lp. plantarum*). The greatest degradation activity was observed by the use of *L. delbrueckii* subsp. *bulgaricus* and *L. plantarum*.

### 3.2. Pickled Vegetables

OPP residual concentrations are, as a rule, high in vegetables. Therefore, pesticide-contaminated fermented vegetable products such as kimchi, sauerkraut, olives, and pickles are correspondingly numerous (Table 3).

The role of lactic acid bacteria in the degradation of chlorpyrifos during *kimchi* fermentation was profoundly investigated [92,93]. Chlorpyrifos was rapidly decreased by day 3 of the fermentation (83.3%), and completely degraded by day 9 [92]. Four species of lactic acid bacteria were isolated and identified as the cause: *Leuconostoc mesenteroides* WCP907, *Lev. brevis* WCP902, *Lp. plantarum* WCP931, and *La. sakei* WCP904. It was found that chlorpyrifos could be used by these four strains as the sole source of carbon and phosphorus [93].

Zhou et al. [94] reported the ability of *Lp. plantarum* to degrade four organophosphate pesticides, including chlorpyrifos, dichlorvos, phorate, and trichlorphon, in sauerkraut and *Mao-tofu*. The results showed that about 16.6–31.8%, 96.2–99.7%, and 79.7–99.5% of the OPPs were degraded after 5 h, 42 h, and 6 days, respectively.

Kumral et al. [95] investigated the degradation potential of two strains of *Lp. plantarum* (LB-1 and LB-2) isolated from fermented black olive brine to eliminate chlorpyrifos (OPP) and deltamethrin (a pyrethroid). LB-1 and LB-2 degraded 96% and 90% of chlorpyrifos and 24% and 53% of deltamethrin in three days, respectively. Maden and Kumral [96] successfully used *Lp. plantarum* 112 (previously isolated from the olive brine) for sauerkraut detoxication from malathion (2 mg/kg) and chlorpyrifos-methyl (4 mg/kg). *Lp. plantarum* strain 123 was efficient in pesticide removal during black olive fermentation, although the process of degradation was relatively slow. At the end of fermentation (after 60 days), 61% deltamethrin, 68% dimethoate, and 50% imidacloprid were removed by the strain [97].

### 3.3. Grains, Flours, and Sourdough

The amylolytic LAB species *Lp. plantarum* is indispensable for reducing pesticide levels in flours, sourdoughs, and silages. Zhang et al. [98] applied *Lp. plantarum* 1.0315, *Lp. plantarum* 1.0624, *Lp. plantarum* 1.0622, and their combination at room temperature for 10 weeks to detoxify corn silage from chlorpyrifos and phorate (0.36 mg/kg). The level of phorate reduction in the treated samples was between 24.9% and 33.4%, depending on the strain. The use of a combination of the three *Lp. plantarum* strains was found to be a more effective strategy in the degradation of OPPs than the use of single strains. Đorđević et al. [99] monitored the degradation of pirimiphos/pirimiphos-methyl by *Lp. plantarum* during wheat fermentation and observed 81% total OPP degradation without any influence on bacterial growth or fermentation activity. Low et al. [100] demonstrated that *Saccharomyces cerevisiae* can degrade glyphosate during bread fermentation, with 21% of the pesticide being degraded within 1 h. Engaging the same yeast species, Sharma et al. [101] reported dissipation of endosulfan (70%), deltamethrin (63%), malathion (60%), propiconazole (52%), chlorpyriphos (51%), and hexaconazole (46%) during dough fermentation (Table 4). However, the role of the starter *Saccharomyces cerevisiae* in the process of detoxication was not elucidated.

An important success was achieved by Đorđević et al. [104] who revealed the possibility to decrease pesticide levels during the fermentation of wheat by yeasts and lactobacilli. When the amounts of pesticides were 15 times above MRL, the degradation rate constants increased by 594% for pirimiphos methyl in the presence of *Saccharomyces cerevisiae*, and 469% for chlorpyrifos due to lactic acid fermentation by *Lp. plantarum*.

### 3.4. Tea, Wine, and Fruit Juices

The only report concerning microbial detoxication of tea is that of Deng et al. [105]. The study proved the degrading ability of the *Aspergillus niger* strain YAT in Chinese brick tea. The strain could degrade 54.83% of β-cypermethrin in 7 days and 100% of 3-phenoxybenzoic acid in 22 h during tea fermentation. These results indicate that the *A. niger* YAT strain has great potential for bioremediation of pyrethroid insecticides in fermented foods. However, due to safety reasons, this is hardly possible.

Multiple pesticide residues were found in grapes and wines, such as fungicides boscalid, penconazole, pyrimethanil, fenhexamid, and iprovalicarb [106]. Actually, 79 different pesticides could be detected in grapes [107], most of them hindering the proper fermentation process of wine and irreversibly changing its aroma [108].

LAB species from the genera *Lactobacillus*, *Leuconostoc*, and *Pediococcus* were found to detoxicate red wine. *Oenococcus oeni*, the most promising species, was able to significantly reduce the concentrations of chlorpyrifos, dicofol, chlorothalonil, and procymidone by 70, 40, 35, and 25%, respectively [109]. Another study by González-Rodríguez et al. [110] reported about 86% decrease in tebuconazole during coupled fermentation of red wine by *Saccharomyces cerevisiae* and *O. oeni*.

Rezaei et al. [111] investigated the ability of probiotic *L. acidophilus* to detoxify apple juice from diazinon (1–5 mg/L). The strain efficiently reduced the pesticide concentration after 72 h and eliminated all traces of it after 28 days of cold storage.

### 3.5. Meat and Sausages

Abou-Arab et al. [112] investigated the effect of starter cultures on the degradation of DDT and lindane during the fermentation of meat products and sausages. When *Lp. plantarum* and *Micrococcus varians* were used as a starter culture and the meat fermentation was prolonged for 72 h, the DDT amount was reduced by 10%, and lindane by 18%.

## 4. Molecular Mechanisms of Pesticides Degradation

Many microbial species involved in food fermentation can metabolize a broad spectrum of pesticides and use them as carbon and energy sources [113]. The major enzymes involved in pesticide degradation belong to the group of phosphoric monoester hydrolases (EC 3.1.3), such as alkaline phosphatase (EC 3.1.3.1), acid phosphatase (EC 3.1.3.2), and phosphoric triester hydrolases (EC 3.1.8), for example, organophosphate hydrolase (OPH, EC 3.1.8.1) and organophosphorus acid anhydrolase (OPAA, EC 3.1.8.2) [87,114]. OPH is a Zn-containing enzyme most effective in hydrolyzing P–O bonds, to a lesser extent also P–F, P–CN, and (least of all) P–S, while OPAA shows 90% similarity with OPH but differs in substrate specificity, being unable to hydrolyze P–S bonds at all, for instance [115]. Carbamates and pyrethroids share with OPP an ester bond in their structure that can be hydrolyzed by esterases. At least 30 esterases with confirmed pesticide-degrading activity have been isolated from plants, animals, and bacteria, but very few of them belong to LAB species traditionally involved in the preparation of fermented foods [116]. Different OPH are encoded by *opd* genes (from organophosphate degrading), while for OPAA synthesis are responsible *opaA* genes [71].

**Figure 3:**
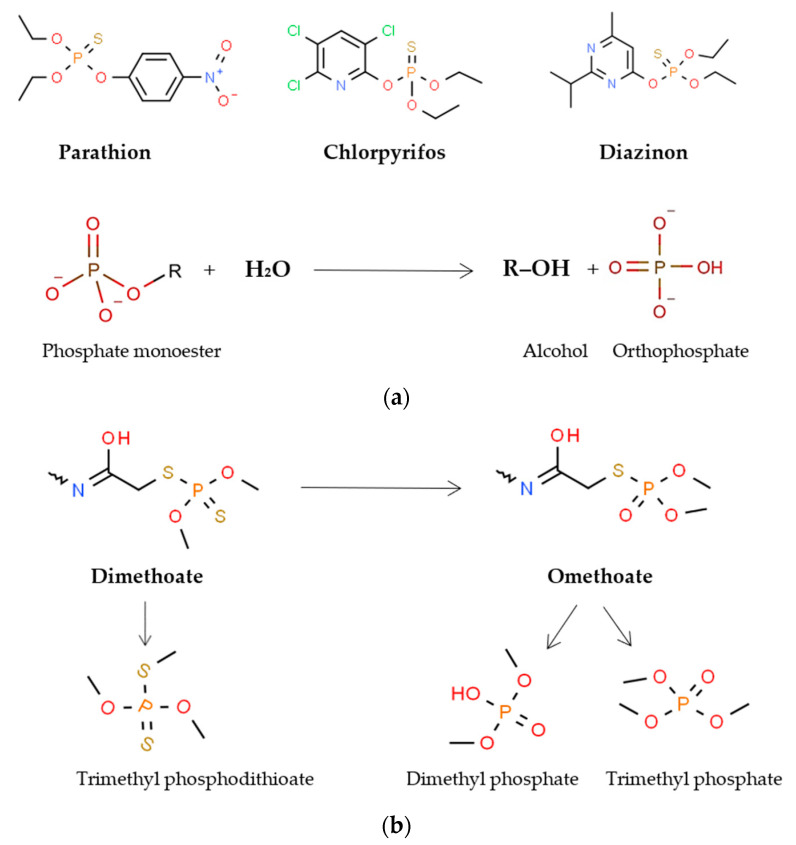
Scheme of the known mechanisms of organophosphate pesticide (shown in bold) degradation by food bacteria. (**a**) Degradation by phosphoric monoester hydrolases such as alkaline phosphatase (EC 3.1.3.1) and acid phosphatase (EC 3.1.3.2) and general chemical equation according to the KEGG database, https://www.kegg.jp/entry/R00626 (accessed on 25 February 2023). (**b**) Dimethoate degradation by phosphatase of *Lp. plantarum* CICC20261. All chemical structures were retrieved from the free chemical structure database ChemSpider, http://www.chemspider.com (accessed on 25 February 2023).

The initial and most important step in the degradation of organophosphate pesticides (OPP) is the hydrolysis of the phosphoesteric (P–O–C) or phosphothiesteric (P–S–C) bond. Three of the most common OPPs, parathion, diazinon, and chlorpyrifos, all share a common P–O–C bond (Figure 3a), which is hydrolyzed to diethylthiophosphoric acid (DETP); *p*-nitrophenol, 2-isopropyl-4-methyl-6-hydroxypyrimidine (IMHP) and 3,5,6-trichloro-2-pyridinol (TCP), respectively [115].

Dialkylphosphate (DAP) metabolites, a group of OPP metabolic products to which DETP belongs, have been associated with increased exposure in recent decades and various neurological pathologies, including impaired intellectual development and attention-deficit disorders in children [117,118,119]. TCP and DETP are toxic and persistent in nature. TCP has been linked with several harmful effects, including reduced testosterone levels in men [120], while DETP has been shown to have a negative influence on sex hormones in women [121]. TCP is known to have an anti-microbial activity that inhibits the growth of chlorpyrifos-degrading microorganisms. Soil bacteria such as *Pseudomonas* and *Enterobacter* can use TCP and DETP as sole sources of carbon, phosphorus, and energy [71,122]. The metabolic fate of TCP and DETP is poorly understood in probiotic strains. Among the relatively few bacteria able to mineralize TCP and DETP, as of yet, none have been confirmed in fermented foods.

Table 5 summarizes the current data concerning the genetic and biochemical characterization of OPP-degrading enzymes of food bacteria.

Relatively few OPP-degrading enzymes in probiotic bacteria have been studied so far in some detail. Five different OP hydrolases (OpdB, OpdD, OpdA, OpdE, OpdC) from four different LAB species (*Lev. brevis*, *La. sakei*, *Leuc. mesenteroides*, *Lp. plantarum*) have been isolated from kimchi, heterologously expressed in *E. coli* and characterized [92,116,117,118]. They all share certain structural features, such as a serine residue critical for their activity and the ‘Gly-X-Ser-X-Gly’ motif typical for serine hydrolases, and they all belong to the GDSVG family of esterolytic enzymes. On the whole, both the original strains in vivo and the recombinant enzymes in vitro show similar abilities for OPP degradation. They are most effective in degrading chlorpyrifos, coumaphos, parathion, methylparathion, and diazinon: well over 50% for nine days at 100 mg/L initial concentration. They are least effective (well below 50% under the same conditions) in degrading dyfonate, cadusafos, ethoprophos, and fenamiphos. The first three of these contain another sulfur atom bound to the phosphorus, that is to say, a P–S–C bond, a somewhat unusual feature that explains at least partly their resistance to OP hydrolases. Fenamiphos has the remnants of an amino group (–NH–) attached to the phosphorus, quite a rare thing for OPP. 

Alkaline phosphatase from *Lc. casei* 355 has been purified and characterized. It has the ability to degrade in vitro the organophosphate insecticide and acaricide dimethoate (1–2 mg/kg) almost in half after four hours that, in combination with its broad tolerance to physical conditions (at least 70% activity at 22–42 °C and pH 7.5–10), makes it a promising candidate for food detoxification [127]. Significant degradation of dimethoate, chlorpyrifos, methylparathion, and trichlorphon (50–87% at 24 h) has also been achieved with *Lp. plantarum* subsp. *plantarum* CICC20261. A positive correlation between this effect and the crude phosphatase activity of the medium was established. Interestingly, however, in vitro degradation by the crude phosphatase was lower and more uniform—around 50% for all four OPP—suggesting an additional mechanism besides the enzymatic degradation, possibly adsorption on the cell surface or perhaps even selective uptake [128]. Phosphatase activity on the level of crude extract has also been confirmed in the degradation of dimethoate by *Lp. plantarum* CICC20261 in milk, which yielded five products of estimated lower toxicity such as omethoate and trimethyl phosphodithioate (Figure 3b), [87]. A positive correlation between phosphatase activity and pesticide degradation was also found for *Lev. brevis* 1.0209 [89] and *L. bulgaricus* [88], but in these cases, not even a crude enzyme was isolated and tested in vitro. Moreover, such statistical methods have been questioned by another study that did not find a significant correlation between OPP degradation and both intracellular and extracellular acid phosphatase activities in *Lp. plantarum* P9 isolated from sour porridge [130].

The experimental design in a number of studies concerned with OPP degradation by probiotic bacteria completely lacks any investigation into the molecular mechanisms. Sometimes the existence of an esterase [92] or an alkaline phosphatase [129] is no more than merely suggested, thus leaving impressive achievements in pesticide detoxification essentially incomplete. Sometimes an adverse effect (i.e., decelerated degradation) is observed, for instance, when *Lp. plantarum* 112 is added as a starter in sauerkraut fermentation [96], but again no mechanism has been suggested.

Pesticide adsorption has been reported for *Lc. casei* WYS3 [126] and *Lc. rhamnosus* GG [82]. Both LAB species showed some ability to sequester chlorpyrifos from the medium. In both studies, adsorption on the cell surface was in some way related to the better studied and more robust mechanism of enzymatic degradation. In the case of *Lc. casei* WYS3, hydrolysis products were detected by GC–MS, and an upregulation of the *opd* gene in the presence of chlorpyrifos was confirmed by RT-PCR. Significantly, *Lc. casei* WYS3 was rather more successful in chlorpyrifos removal: 80% after four days at 50 mg/L initial concentration. An OP hydrolase was predicted in the genome of *Lc. rhamnosus* GG but was not found to be functional. Compared to enzymatic degradation, pesticide adsorption is a less effective way of detoxification and seems less likely to engender any scientific breakthroughs in the future.

Microbial degradation of organochlorine pesticides [131], carbamates [132], and ne-onicotinoids [133] has been studied extensively for decades. A number of bacterial species isolated from soil and water have been implicated in the process. As of yet, however, there are no probiotics from fermented foods among them. Considering the remarkable results achieved with such bacteria in the degradation of organophosphates, their application for biodegradation of other pesticides may prove to be an exciting area of future research. 

## 5. Prospects in Fermented Food Detoxification

Pesticides are a threat to human health of global magnitude. Chlorpyrifos, for example, is found in fruits and vegetables all over the world, from cucumbers in Thailand (275 μg/kg) to apples in Slovakia and Poland (21–93 μg/kg), even though in all three countries, the use of chlorpyrifos is banned [134]. Some fermented foods can be detoxified from pesticides thanks to the activity of the bacterial microflora present. Lactic acid bacteria are known for their antagonistic activity against diseases caused by fungi and for which plants are treated with tons of fungicides [135,136,137,138,139]. In these cases, the biological approach should be preferred to chemical methods of pesticide removal. On the other hand, the selection of strains with detoxification potential can occur in natural habitats. For example, LAB isolated from the gut of bees exposed to pesticides such as chlorpyrifos, coumaphos, and imidacloprid are capable of binding and neutralizing them in vitro as well [140].

Functional foods rich in probiotic LAB have the potential of combating accidentally ingested pesticides in the GIT directly, by degradation or absorption as already discussed, or indirectly by neutralizing the adverse effects of pesticides [15]. Many lactobacilli have potent antioxidant properties and may be able to alleviate the oxidative stress and damage caused by chronic exposure to OPP [141]. One recent study showed the antioxidant capacity of *Lp. plantarum* Pb3 is increased in the presence of chlorpyrifos, imidacloprid (a neonicotinoid), and chlorantraniliprole (an insecticide from the ryanoid class). A high survival rate (70–75%) in simulated gastric and intestinal juices also makes the strain a suitable candidate for combating the adverse effects of ingested pesticides. It should be noted, however, that both the ability to inhibit lipid peroxidation and to scavenge hydroxyl radicals were slightly increased (5–10%) in the presence of the pesticides [142]. In vivo studies with rats show that *Lp. plantarum* BJ0021 can alleviate most harmful effects of endosulfan (an organochlorine insecticide and acaricide) yet has no positive effect on major antioxidant enzymes such as SOD and CAT [81]. The antioxidant properties of LAB should be treated with caution.

Another indirect influence is the ability of many LAB species to enhance the gut barrier function and thus prevent the absorption of pesticides. This is only one of the numerous beneficial effects of LAB on intestinal health that are supported by a great and growing body of evidence [15,143,144]. *Lp. plantarum* MB452 has been shown to affect the expression of 19 genes related to tight junctions, thus improving the integrity and signaling in human colon cancer (Caco-2) cells, a common model for intestinal epithelium [145]. *Lc. rhamnosus* strains GG and GR-1 reduced the absorption of 100 μM parathion and especially chlorpyrifos within 60 min in Caco-2 Transwell model of the small intestine epithelium [82]. Far from being merely preventive medicine, probiotic bacteria can also help once the damage is done. A cocktail of four *Lactobacillus* species (JUP-Y4) isolated from traditional Chinese fermented foods was shown to improve the recovery of antibiotic-induced intestinal disruption in mice, including enhanced integrity of the gut barrier, reduced inflammation, lower levels of endotoxins in the blood, and restored numbers of beneficial gut bacteria [146].

Neurodegenerative diseases, especially Parkinson’s disease, have also been linked with pesticide exposure and, consequently, probiotic bacteria may add to their health benefits a neuroprotective effect [147]. In addition to pesticides, probiotics are studied as a potential weapon against many other toxic substances [148]. Innovative microbial processes are developed with the certain potential to detoxify foods from mycotoxins, polycyclic aromatic hydrocarbons, perfluoroalkyl and polyfluoroalkyl compounds, phthalates, bisphenol A, and heavy metals.

## 6. Conclusions

Although pesticides are indispensable chemical agents widely used in agriculture to increase yields of key crops for human nutrition and to control pests, significant residual amounts of them are found in foods of plant and animal origin. Besides physical detoxification methods, a very successful and promising approach is fermentation by natural microflora in foods or by purposefully added probiotic strains. Detoxification mechanisms include enzymatic hydrolysis or oxidation of pesticides, which are degraded to less poisonous products, and intermediate metabolites have been demonstrated in some scientific publications by analysis with various analytical methods. In most cases, however, the exact metabolic pathway of degradation by the strains in food has not been elucidated and requires further research in the future. The application of microbial food detoxification is of particular importance in less developed countries where control of pesticide residues is weaker. In addition, naturally fermented plant-based products are common as traditional ethnic foods in these countries.

## Figures and Tables

**Table 1:** Pesticides that are found in foods in concentrations highly exceeding the MRL.

Food	Pesticide Name	Pesticide Class	Toxic Effects on Human Health	Content * (mg/kg)	Reference
Wheat	Flutriafol	Fungicide	Liver function disorders	0.350	[31]
	Chlorpyrifos	Insecticide	Headache, blurred vision, coma, death	0.338	[32]
	Cypermethrin	Insecticide	Irritation to the skin, numbness, tingling, incoordination, death	0.058	[33]
Maize	Metribuzin	Herbicide	Stomach aches, fatigue, depression, disturbance in kidneys	1.390	[34]
	Thiofanox	Insecticide	Cancerogen	0.083	[33]
Chinese cabbage	Chlorpyrifos	Insecticide Insecticide Insecticide Insecticide	Headache, blurred vision, coma, death	0.48	[35]
	Bifenthrin		Endocrine disrupting effects	1.19	[36]
	Methoxyfenozide		Liver, and thyroid systems toxicity	1.12	[37]
	Pymetrozine		Causes diabetes	3.02	[38]
Tomato	Chlorates	Herbicide	Gastritis, toxic nephritis, hemolysis, methemoglobinemia, hemoglobinuria	0.450	[39]
Spinach	Methamidophos	Insecticide	Nausea, vomiting, weakness, paralysis	2.948	[40]
Peppers	Chlordecone	Insecticide	Carcinogenic, with neurological, reproductive, and developmental toxicity	0.510	[41]
Eggplant	Carbaryl	Insecticide	Sweating, nausea, vomiting, abdominal pain	1.052	[42]
Potato	Kresoxim	Fungicide	Carcinogenic	0.180	[43]
Peach	Carbofuran	Insecticide	Endocrine system disruptor	1.660	[44]
Leaf mustard	Deltamethrin	Insecticide	Headaches and dizziness	0.661	[45]
Chinese kale	Cypermethrin	Insecticide	Numbness, incoordination, death	8.79	[46]
Groundnuts	Cymoxanil	Fungicide	Lungs hyperplasia, gliosis, spongiosis	1.46	[34] [34]
Egusi seeds	Atrazine	Herbicide	Reproductive system disorders	1.41	

* The highest values reported in the respective food on the market.

**Table 2:** The main microbial species found to reduce pesticide levels in milk, yogurt, and cheeses.

Source	Species and Strain	Pesticide	Reduction	Reference
Egypt Ras cheese, milk	*Lactobacillus delbrueckii* subsp. *bulgaricus,* *Streptococcus thermophilus,* yeasts	DDT	40.6% at 0.1 mg/kg fat 33.9% at 1.0 mg/kg fat 25.5% at 10.0 mg/kg fat	[73]
Yogurt, cheese	*Lactobacillus acidophilus, L. delbrueckii* subsp. *bulgaricus*, *Lactiplantibacillus plantarum*, *Lacticaseibacillus rhamnosus*, *Lacticaseibacillus casei*, *S. thermophilus*, *Bifidobacterium bifidum*	α-hexachlorocyclohexane (HCH), hexachlorobenzene (HCB), γ-HCH, β-chlordane, α-chlordane	37.0–50.9% after 12 h at 20 μg/kg for all	[85]
Fermented beverages from cow and goat milk (bio-yogurts)	*L. acidophilus* LA-5, *Bif. animalis* subsp. *lactis* BB-12	αHCH, βHCH, γHCH, 1,1-bis-(4-chlorophenyl)-2,2-dichloroethene (pp’DDE), 1-chloro-4-[2,2-dichloro-1-(4-chlorophenyl) ethyl] benzene, 1,1′-(2,2,2-trichloroethane-1,1-diyl) bis(4-chlorobenzene)	up to 48.6% (heptachlor) and 54.7% (pp’DDE) in goat milk bio-yogurts after 14 days of cold storage when both cultures were used (synergistic effect)	[86]
Yogurt	*L. delbrueckii* subsp. *bulgaricus,* *S. thermophilus*	Dimethoate, fenthion, malathion, methyl parathion, monocrotophos, phorate, trichlorphon	9.2–17.1% after 4 h at 1.5 mg/kg for all except malathion	[77]
Milk	*L. plantarum* CICC20261	Dimethoate	81.28% at 50 mg/kg for 24 h	[87]
Skimmed milk	*L. delbrueckii* subsp. *bulgaricus*, *L. acidophilus*, *Lc. casei, Lc. rhamnosus, S. thermophilus*	Chlorpyrifos, chlorpyrifos-methyl, diazinon, dichlorvos, fenthion, malathion, phorate, pirimiphos-methyl, trichlorphon	up to 64.6% for dichlorvos with *L. bulgaricus* at 1 mg/kg for 24 h	[88]
Skimmed milk	*Lp. plantarum* strains 1.0317, 1.0624, 1.0315, 1.066, *Levilactobacillus brevis* 1.0209, *Lactobacillus helveticus* strains 1.0203 and 1.9204, *Lactobacillus lactis* 4.0611, *L. delbrueckii* subsp. *bulgaricus* L6, *S. thermophilus* 3.0503	Chlorpyrifos, diazinon, fenitrothion, malathion, methyl parathion	≈50% at 0.6 mg/kg diazinon for 24 h with *Lev. brevis* 1.0209	[89]
Skimmed milk	*L. bulgaricus, Lacticaseibacillus paracasei, Lp. plantarum*	Dimethoate, fenthion, malathion, methyl parathion, monocrotophos, phorate, trichlorphon	from 20.9% (methyl parathion with *Lc. paracasei*) to 46.9% (malathion with *Lp. plantarum*) at 1.2 mg/kg for 24 h	[90]

**Table 3:** The main microbial species and strains found to reduce pesticide levels in pickled vegetables.

Source	Species and Strain	Pesticide	Reduction	Reference
*Kimchi*	*Leuconostoc mesenteroides* WCP907, *Lev. brevis* WCP902, *Lp. plantarum* WCP931, *La. sakei* WCP904	Chlorpyrifos, coumaphos, diazinon, parathion, methyl parathion	100% chlorpyrifos for 9 days at 200 mg/L initial concentration	[92]
*Kimchi*	*Lev. brevis* WCP902	Chlorpyrifos, coumaphos, diazinon, parathion, methyl parathion	≈75% chlorpyrifos for 9 days at 100 mg/L initial concentration	[93]
Pickled Chinese cabbage	*Lp. plantarum*	Chlorpyrifos, dichlorvos, phorate, trichlorphon	6.5–18% more compared to normal fermentation after 42 days at 1 mg/kg; <1% of all except chlorpyrifos (≈4%) in the end	[94]
Black olives	*Lp. plantarum* (LB-1 and LB-2)	Chlorpyrifos, deltamethrin	96% for chlorpyrifos after 3 days and 86% for deltamethrin after 10 days for LB-1 at 100 mg/L	[95]
Sauerkraut	*Lp. plantarum* 112	λ-cyhalothrin, malathion, chlorpyrifos-methyl	13–19% for λ-cyhalothrin (2 mg/kg) after 14 days; 34–59% for the other two (2–4 mg/kg), but lower than natural fermentation (69–98%)	[96]
Black olives	*Lp. plantarum* 112, *Lp. plantarum* 123	Deltamethrin, dimethoate, imidacloprid	2–8% greater compared to natural fermentation after 60 days at 25–350 g/L initially	[97]

**Table 4:** The main microbial species and strains found to reduce pesticide levels in flour, bread, and sourdough.

Source	Species and Strain	Pesticide	Reduction	Reference
Corn silage	*Lp. plantarum* 1.0315, *Lp. plantarum* 1.0624, *Lp. plantarum* 1.0622	Chlorpyrifos, phorate	33.4% for phorate (0.36 mg/kg) after 10 weeks, but very close to control fermentation (26.2%)	[98]
Bread wheat	*Lp. plantarum*	Pirimiphos, pirimiphos-methyl	15–34% pirimiphos-methyl (5 mg/kg) for 48 h	[99]
Bread	*Saccharomyces cerevisiae*	Glyphosate	21% for 1 h at 50 mg/kg	[100]
Bread	*Saccharomyces cerevisiae*	Endosulfan, deltamethrin, malathion, propiaconazole, chlorpyriphos, hexaconazole	75–89% at 1 mg/kg; 47–70% at 4 mg/kg; after 12 h fermentation at 30 °C and 20 min baking at 80 °C	[101]
Wheat flour	*Lp. plantarum*	Bifenthrin	42% at 0.5 mg/kg, only 18% at 2.5 mg/kg	[102]
Alfalfa silage	*Lp. pentosus* 3–27	Beta-cypermethrin	96% at 50 mg/L for 4 days	[103]
Wheat	*Saccharomyces cerevisiae*	Pirimiphos methyl	48.8% at 5 mg/kg for 72 h	[104]
Wheat	*Lp. plantarum*	Chlorpyrifos methyl	56.7% at 3 mg/kg for 72 h	[105]

**Table 5:** Enzymatic hydrolysis of organophosphate pesticides.

Pesticide	Species/Strain	Acting Enzyme	Gene	Protein	Features	Reference
Chlorpyrifos, methylparathion, parathion, coumaphos, diazinon	*Lev. brevis* WCP902	Organophosphate hydrolase	*opdB* 723 bp	240 AA 27 kDa	‘Gly-X-Ser-X-Gly’-motif; Ser82 * pH 6.0, 35 °C	[93]
	*La. sakei* WCP904	Organophosphate hydrolase	*opdD* 825 bp	274 AA 31 kDa	‘Gly-X-Ser-X-Gly’-motif; Ser 116 * pH 6, 30 °C	[123]
	*Leuc. mesenteroides* WCP307	Organophosphate hydrolase	*opdA* 930 bp	309 AA 35 kDa	‘Gly-X-Ser-X-Gly’-motif; Ser128 *; pH 7, 30 °C	[124]
	*Leuc. mesenteroides* WCP307	Organophosphate hydrolase	*opdE* 894 bp	297 AA 33 kDa	‘Gly-X-Ser-X-Gly’ -motif, Ser129 *; pH 6, 30 °C	[124]
	*Lp. plantarum* WCP931, *Leuc. mesenteroides* WCP907, *Lev. brevis* WCP902, *La. sakei* WCP904	Esterase (suggested)	-	-	-	[92]
Parathion, methylparathion, chlorpyrifos	*Lp. plantarum* WCP931	Organophosphate hydrolase	*opdC* 831 bp	276 AA 31 kDa	‘Gly-X-Ser-X-Gly’—motif; Ser 116 *; pH 6, 35 °C	[125]
Chlorpyrifos	*Lc. casei* WYS3	Organophosphate hydrolase	*opda*	-	(+) *opda* RNA levels; hydrolysis products detected by GC-MS	[126]
Dimethoate	*Lc. casei* 355	Alkaline phosphatase	-	43 kDa	pH 8.5, 37 °C (+) Mg^2+^, Ca^2+^	[127]
		(purified)			(−) Cu^2+^, Zn^2+^, EDTA	
	*Lp. plantarum* CICC20261	Phosphatase (crude activity)	-	-	Gln375 and Ser415 *	[87]
Dimethoate, chlorpyrifos, trichlorphon, methylparathion	*Lp. plantarum* subsp. *plantarum* CICC20261	Phosphatase (crude activity)	-	-	-	[128]
Trichlorphon, phorate, malathion, dichlorvos	*L. delbrueckii* subsp. *bulgaricus, S. thermophilus, Lp. rhamnosus*	Phosphatase activity (correlation)	-	-	-	[88]
Chlorpyrifos, fenitrothion, malathion	*Lev. brevis* 1.0209	Phosphatase activity (correlation)	-	-	-	[89]
Chlorpyrifos	*Pediococcus pentasaceus* 4320, *Leuc. mesenteroides* 8293, *Ent. faecium* 86, *Lactococcus. lactis* 1454, *Lc. rhamnosus* GG53103, *Leuc. lactis* 19256	Alkaline phosphatase (suggested)	-	-	-	[129]

* Amino acid residue in the active center, which is crucial for enzyme hydrolysis.

## Data Availability

The data presented in this study are available on request from the corresponding author.
